# Macrophage Dicer promotes tolerogenic apoptotic cell clearance and immune tolerance by inhibiting pentose phosphate pathway activity

**DOI:** 10.1038/s41423-021-00693-w

**Published:** 2021-05-18

**Authors:** Qiangdongzi Mao, Bangwei Luo, Jie Mei, Wenhua Li, Xue Zhang, Zhiyu Wang, Jiechun Zhang, Tingting Liu, Fengxue Zhang, Zhiren Zhang

**Affiliations:** 1 Research Center for Integrative Medicine of Guangzhou University of Chinese Medicine, Guangzhou, Guangdong China; 2 Institute of Immunology, Army Medical University, Chongqing, China; 3 Department of Respiratory Medicine, The First Affiliated Hospital of Guangzhou University of Chinese Medicine, Guangzhou, Guangdong China

**Keywords:** Autoimmunity, Monocytes and macrophages

Given that apoptosis is the dominant modality of homeostatic cell turnover, efficient clearance of apoptotic cells (ACs), which is a process known as efferocytosis, is critical for immune homeostasis. The phagocytic engulfment of ACs occurs through an immunologically silent process to prevent an immune response to self-antigens. Correspondingly, defects in the removal of ACs have been proposed to underlie the pathogenesis of systemic lupus erythematosus (SLE).^1,2^ However, pathways that regulate phagocytes for efficient immune-silent AC uptake remain to be fully elucidated. Dicer is best known for its role as a riboendonuclease in the biogenesis of microRNAs.^3^ While recent studies have determined a link between microRNAs and AC clearance, the interplay between Dicer and efferocytosis remains unknown.^4^

We first examined whether macrophage Dicer contributed to AC clearance. Apoptotic thymocytes were labeled with a pH-sensitive dye (pHrodo) to distinguish between engulfed and bound cells (Fig. S1A). Peritoneal macrophages (PMs) or bone marrow-derived macrophages from *Dicer1-CKO* (*LysM-Cre*^*+/+*^/*Dicer1*^*loxp/loxp*^) mice exhibited significant reductions in AC phagocytosis compared to their respective WT or *Dicer1-C* (*LysM-Cre*^*+/+*^/*Dicer1*^*+/+*^) controls in vitro (Figs. 1A, S1B). Then, we used the classic in vivo dying thymocyte clearance assay. Following dexamethasone stimulation, thymi of WT mice showed marked loss of mass and few Annexin V^+^ cells. In contrast, despite similar recruitment of CD68^+^ macrophages, thymi of *Dicer1-CKO* mice contained increased numbers of free Annexin V^+^ cells and higher mass than WT control thymi (Fig. 1B). Moreover, Dicer deficiency enhanced the macrophage inflammatory response during AC clearance, as reflected by increased expression of TNF-α, IL-1β, and IL-6 and decreased levels of TGF-β and IL-10 compared to those of WT macrophages (Fig. 1C).

**Fig. 1:**
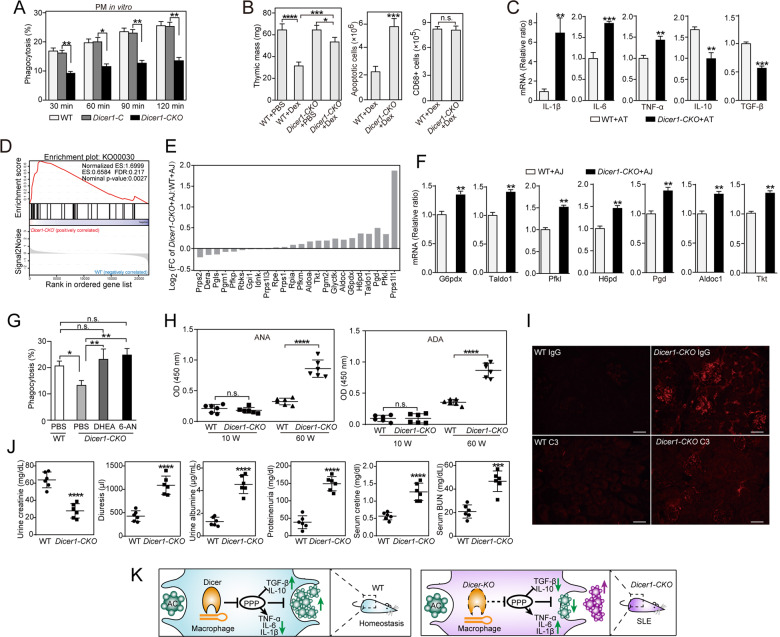
Dicer regulates immune-silent clearance of ACs and immune tolerance by inhibiting pentose phosphate pathway activity in macrophages. **A** In vitro cultured WT, *Dicer1-C* or D*icer1-CKO* mouse peritoneal macrophages were incubated with pHrodo-labeled apoptotic thymocytes for the indicated times, and phagocytosis was analyzed by flow cytometry (*n* = 3). **B** Dexamethasone (Dex, 0.2 mg) was i.p. injected into 4-week-old WT or *Dicer1-CKO* mice, and after 24 h, the numbers of Annexin V+ apoptotic cells and CD68+ macrophages were analyzed by flow cytometry (*n* = 3). **C** In vitro cultured WT or *Dicer1-CKO* mouse peritoneal macrophages were incubated with apoptotic thymocytes for 24 h, and the mRNA expression of inflammatory cytokines in macrophages was measured by quantitative RT-PCR (*n* = 3). **D**–**F** In vitro cultured WT or *Dicer1-CKO* mouse peritoneal macrophages were incubated with apoptotic human Jurkat T cells for 6 h, and macrophage transcriptional profiles were assessed by mRNA sequencing (**D**, **E** *n* = 3) or quantitative RT-PCR (**F**, *n* = 3). **D** KEGG enrichment plot of the pentose phosphate pathway (KO00030), as determined by Gene Set Enrichment Analysis (GSEA). **E** The expression of enriched genes in the pentose phosphate pathway, as determined by GSEA. **F** Validation of enriched genes in the pentose phosphate pathway by quantitative RT-PCR. **G** In vitro cultured WT or *Dicer1-CKO* mouse peritoneal macrophages were incubated with DHEA (1 μM), 6-AN (10 μM) or PBS for 24 h, and the phagocytosis of pHrodo-labeled apoptotic thymocytes was measured by flow cytometry (*n* = 3). **H** Serum concentrations of anti-dsDNA antibodies (ADAs) and anti-nuclear antibodies (ANAs) in WT and *Dicer1-CKO* mice of different ages were analyzed by ELISA (*n* = 6). **I** IgG and C3 levels in the kidneys of 60-week-old WT and *Dicer1-CKO* mice (*n* = 3, bar = 100 μm). **J** Kidney functions in 60-week-old WT and *Dicer1-CKO* mice (*n* = 6). **K** Schematic diagram of the role of macrophage Dicer in apoptotic cell clearance and immune tolerance. The results are expressed as the mean ± SEM, n.s. not statistically significant, *P < 0.05; **P < 0.01; ***P < 0.001; ****P < 0.0001, two-tailed Student’s *t* test for two groups (**B**, **C**, **F**, **J**), one-way (**B**, **G**, **H**) or two-way (**A**) ANOVA with Tukey’s post hoc test for multiple groups

To explore the mechanisms underlying Dicer-mediated AC removal, we compared the transcriptional profiles of WT or Dicer-KO PMs that were incubated with ACs by mRNA sequencing. Gene set enrichment analysis identified significant that the pentose phosphate pathway (PPP) gene signature was substantially enriched in Dicer-KO macrophages but not in WT cells (Figs. 1D, E, S2A). Furthermore, the levels of seven PPP-associated genes and their products nicotinamide adenine dinucleotide phosphate (NADPH) and glutathione^5^ were significantly higher in Dicer-KO PMs than in WT macrophages during efferocytosis (Figs. 1F, S2B, C). In addition, even without AC stimulation, Dicer deficiency alone increased the expression of PPP-related genes (Fig. S2D) and the levels of NADPH and glutathione (Fig. S2E) in PMs. The PPP antagonists dehydroepiandrosterone (DHEA) and 6-aminonicotinamide (6-AN)^6^ blocked PPP activity (Fig. S2F), restored AC phagocytosis to WT levels (Fig. 1G) and reduced the inflammatory response during efferocytosis (Fig. S2G) in Dicer-KO macrophages in vitro. These data indicate that Dicer modulates tolerogenic AC removal mainly through the PPP in macrophages.

The efficient clearance of ACs is essential for immune tolerance; therefore, we examined whether *Dicer1-CKO* mice developed spontaneous lupus-like symptoms. In self-generated *Dicer1-CKO* mice, the Dicer level was reduced by 93% in macrophages (Fig. S3A–C). Significantly higher accumulation of ACs was observed in 60-week-old but not 10-week-old *Dicer1-CKO* mice than in their respective WT controls (Fig. S3D–F). In addition, *Dicer1* deficiency did not enhance neutrophil or macrophage apoptosis in vitro compared to that of their respective WT counterparts (Fig. S3G). In line with the increase in AC deposition, 60-week-old *Dicer1-CKO* mice exhibited significant increases in serum levels of anti-dsDNA antibodies and anti-nuclear antibodies (Fig. 1H), prominent IgG and C3 deposition in the glomeruli of kidneys (Fig. 1I), impaired kidney functions, as reflected by increased serum levels of creatinine and blood urea nitrogen (Fig. 1J), and increases in inflammatory cell infiltration in multiple tissues (Fig. S3H) compared to those of matched WT counterparts. Briefly, these data demonstrate that genetic *Dicer1* deficiency in myeloid cells results in the development of SLE-like disease.

This work provides several new insights into the understanding of immune-silent AC clearance and the maintenance of immune tolerance (Fig. 1K). Our investigation reveals an essential role of Dicer in increasing tolerogenic AC phagocytosis. Our work also provides the first evidence for the involvement of the PPP in regulating immune-silent AC clearance in macrophages. Finally, our data identify a previously unknown link between macrophage Dicer and immune tolerance in mice.

## Supplementary information

supplemental figure and figure legend

supplemental material method
