# Supplementary material for: Macrophage Dicer promotes tolerogenic apoptotic cell clearance and immune tolerance by inhibiting pentose phosphate pathway activity
Source: Cell Mol Immunol. 2021 May 18;18(7):1841–3. doi: 10.1038/s41423-021-00693-w (PMC8245631; doi:10.1038/s41423-021-00693-w)
Supplement: Supplementary file 1 — supplemental figure and figure legend [file 41423_2021_693_MOESM1_ESM.docx]

**Macrophage Dicer promotes tolerogenic apoptotic cell clearance and immune tolerance through inhibiting pentose phosphate pathway activity**


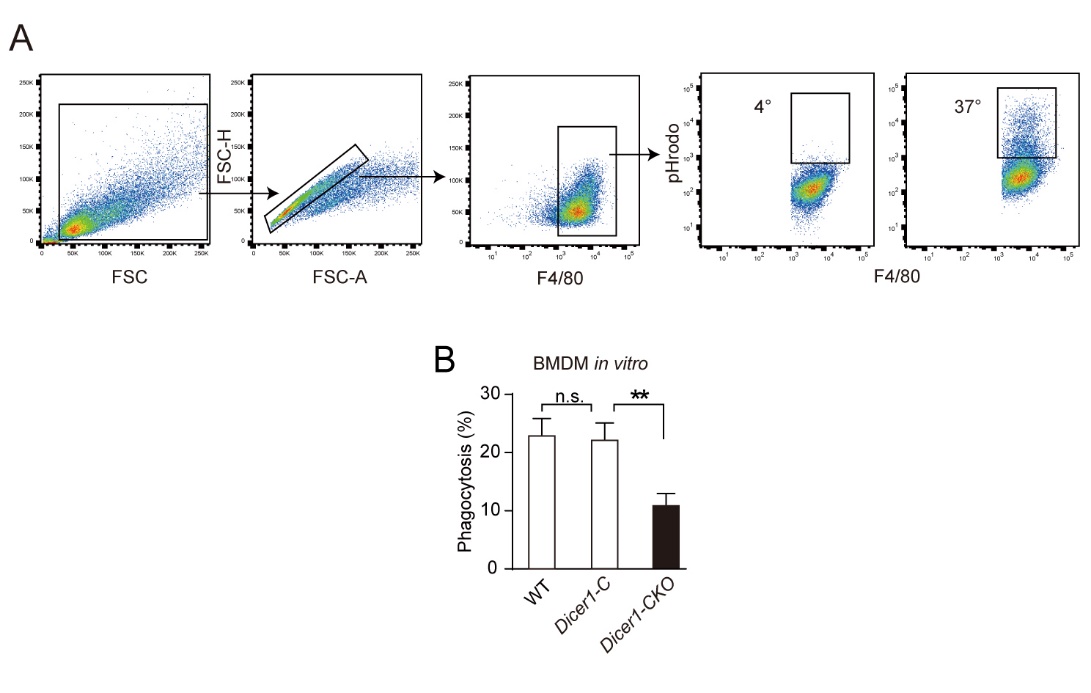


**Figure S1. Dicer regulates immune-silent clearance of ACs by macrophages.**

**A**: *In vitro* cultured mice peritoneal macrophages were incubated with pHrodo labelled apoptotic thymocytes for 60 min, engulfment of apoptotic thymocytes by macrophages was detected by flowcytometry, gating strategy was shown. **B**: *In vitro* cultured WT, *Dicer1-C* or *Dicer1-CKO* mice bone marrow-derived macrophages (BMDMs) were incubated with pHrodo labelled apoptotic thymocytes for 60 min, phagocytosis of apoptotic thymocytes by BMDMs was assayed by flowcytometry (n = 3). Result was expressed as mean ± SEM, n.s.: not statistically significant, ***P* < 0.01 (one-way ANOVA with Tukey’s post hoc test for multiple groups).


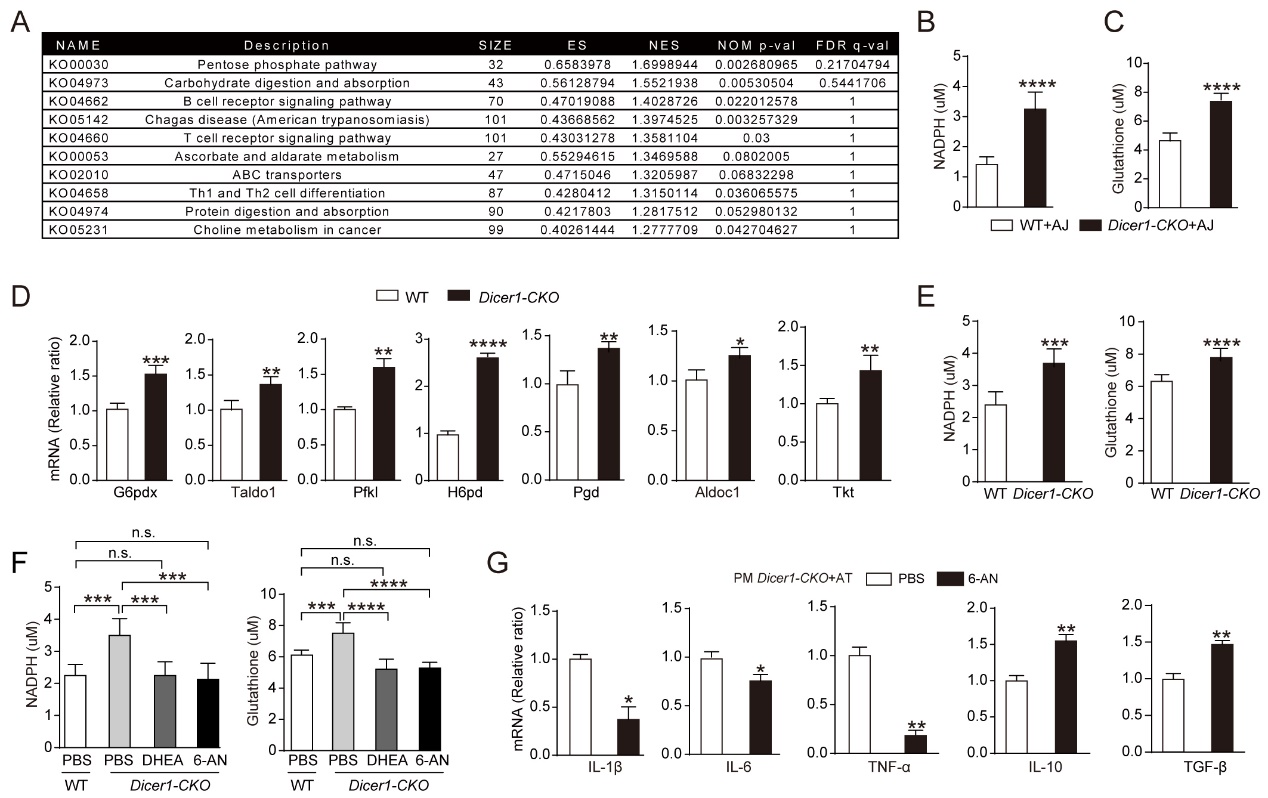


**Figure S2. Dicer modulates AC clearance through pentose phosphate pathway in macrophages.**

**A**: *In vitro* cultured WT or *Dicer1-CKO* mice peritoneal macrophages were incubated with apoptotic human Jurkat T cells for 6 hours, macrophage transcriptional profiles were detected by mRNA sequencing (n = 3). Top 10 of enrichment results by Gene Set Enrichment Analysis (GSEA) were shown. **B-C**: *In vitro* cultured WT or *Dicer1-CKO* mice peritoneal macrophages were incubated with apoptotic human Jurkat T cells for 24 hours, levels of nicotinamide adenine dinucleotide phosphate (NADPH) and glutathione in peritoneal macrophages were detected by ELISA (n = 6). **D-E**: WT and *Dicer1-CKO* mice peritoneal macrophages were *in vitro* cultured, pentose phosphate pathway genes (**D**, n = 3), levels of NADPH and glutathione (**E**, n = 6) were detected. **F**: *In vitro* cultured WT or *Dicer1-CKO* mice peritoneal macrophages were incubated with DHEA (1 μM), 6-AN (10 μM) or PBS for 24 hours, levels of NADPH and glutathione in macrophages were detected by ELISA (n = 6). **G**: *In vitro* cultured *Dicer1-CKO* mice peritoneal macrophages were incubated with apoptotic thymocytes in presence of 6-AN (10 μM) or PBS for 24 hours, mRNA levels of inflammatory cytokines in macrophages were measured by quantitative RT-PCR (n = 3). Results were expressed as mean ± SEM, n.s.: not statistically significant, **P* < 0.05, ***P* < 0.01, ****P* < 0.001 and *****P* < 0.0001, two-tailed Student’s t-test (**B**, **C**, **D**, **E**, **G**) or one-way ANOVA with Tukey’s post hoc test for multiple groups (**F**).


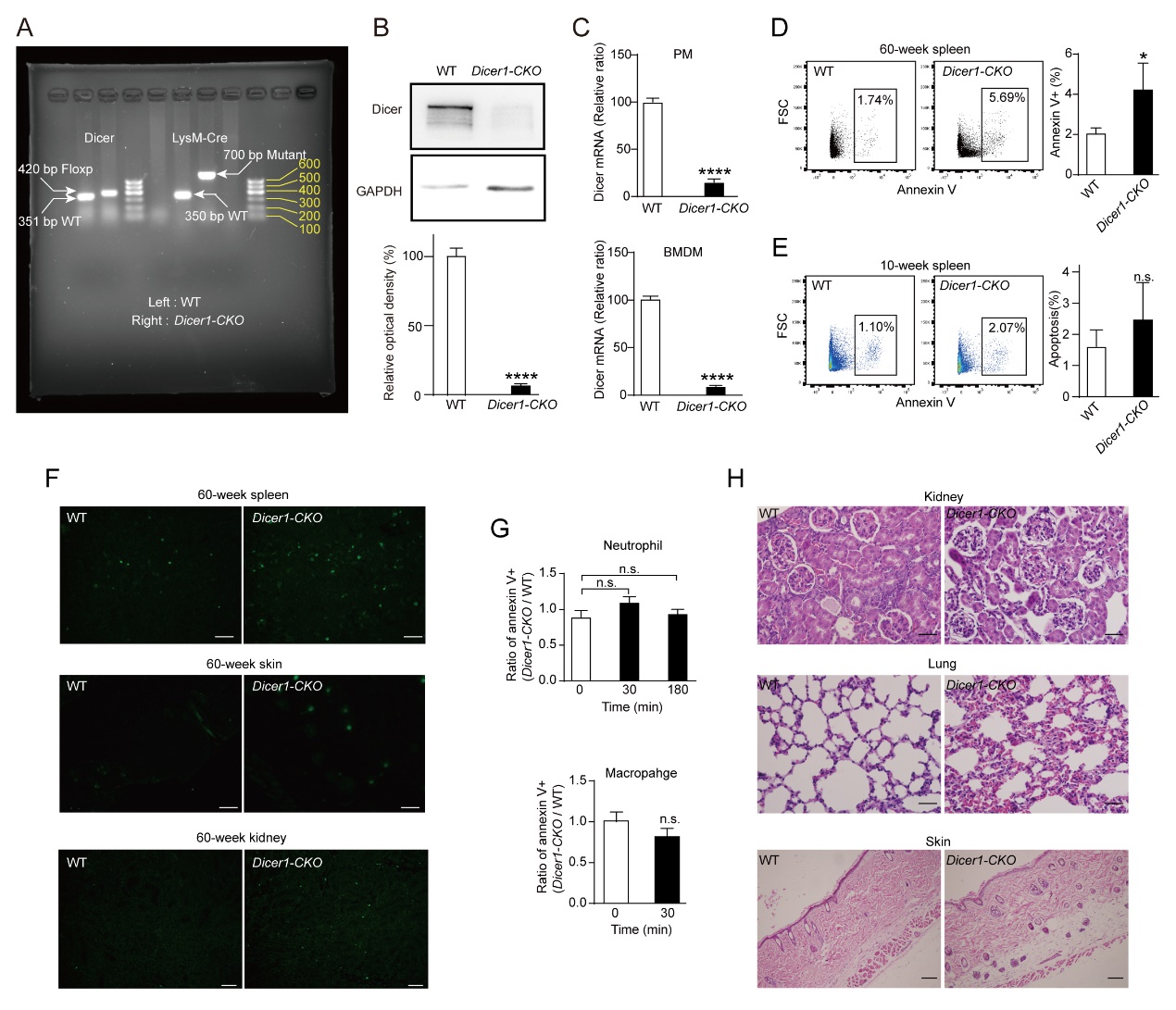


**Figure S3. Myeloid Dicer-deficient mice develop systemic autoimmune disease.**

**A**: The identification of PCR products by electrophoresis. **B**: Western blot detection of Dicer protein in WT and *Dicer1-CKO* peritoneal macrophages (n = 3). **C**: Quantitative RT-PCR detection of Dicer mRNA in WT and *Dicer1-CKO* peritoneal macrophages and BMDMs (n = 3). **D-E**: Apoptotic cell accumulation in 60-week-old and 10-week-old WT and *Dicer1-CKO* mice spleen detected by flowcytometry (n = 3). **F**: TUNEL staining of apoptotic cells in tissues of spleen, skin and kidney from 60-week-old WT and *Dicer1-CKO* mice (n = 3, bar = 100 μm). **G**: *In vitro* cultured WT or *Dicer1-CKO* mice neutrophils were stimulated for 30 or 180 min, neutrophil apoptosis was detected by flowcytometry (n = 3); *In vitro* cultured WT and *Dicer1-CKO* mice peritoneal macrophages were stimulated with dexamethasone (10 μM) for 30 min, apoptosis was detected by flowcytometry (n = 3). **H**: HE staining of kidney, lung and skin tissues of WT and *Dicer1-CKO* mice (n = 3, bar = 100 μm). Results were expressed as mean ± SEM, n.s.: not statistically significant, **P* < 0.05 and *****P* < 0.0001, two-tailed Student’s t-test for two groups (**B**, **C**, **D**, **E**, **G**) or one-way ANOVA with Tukey’s post hoc test for multiple groups (**G**).
