# Supplementary material for: Macrophage Dicer promotes tolerogenic apoptotic cell clearance and immune tolerance by inhibiting pentose phosphate pathway activity
Source: Cell Mol Immunol. 2021 May 18;18(7):1841–3. doi: 10.1038/s41423-021-00693-w (PMC8245631; doi:10.1038/s41423-021-00693-w)
Supplement: Supplementary file 2 — supplemental material method [file 41423_2021_693_MOESM2_ESM.docx]

**Macrophage Dicer promotes tolerogenic apoptotic cell clearance and immune tolerance through inhibiting pentose phosphate pathway activity**

**Materials and methods**

**Animals.**

All mice were housed under pathogen-free conditions in the animal facility of Army Medical University. Experiments were performed according to local ethical guidelines and were approved by the local Administration District Official Committee of Army Medical University, Chongqing, China. C57BL/6 mice were purchased from Vital River Laboratories, Beijing, China. Mice carrying the lox-P-targeted *Dicer1* (*Dicer1^loxp/loxp^*) allele and the *lysozyme-M Cre* (*LysMCre*) recombinase transgene were purchased from the Jackson Laboratory. *Dicer1^loxp/loxp^* and *LysMCre* mice were crossed to generate myeloid-specific *Dicer1* knockout mice lacking macrophage *Dicer1* expression. We refer to *Dicer1^loxp/loxp^LysMCre^+/+^* littermates as *Dicer1-CKO* mice and *Dicer1^-/-^LysMCre^+/+^* littermates as *Dicer1-C* mice. We assessed spontaneous autoimmunity development in female *Dicer1-CKO* mice at age of 10 and 60 weeks.

**Generation of apoptotic cells.**

Thymocytes were harvested from 4- to 6-weeks old female C57BL/6 mice and cultured in RPMI (Gibco BRL, Grand Island, NY, USA) supplemented with 10% foetal bovine serum (FBS, Gibco BRL, Grand Island, NY, USA). To generate apoptotic thymocytes, cells were treated with 1 mM dexamethasone (Sigma-Aldrich Corp, Darmstadt, Germany) for 4-6 hours at 37°C. Afterwards, cells were washed three times with HBSS (Gibco BRL, Grand Island, NY, USA) and resuspended in 10% FBS in RPMI. The apoptosis of Jurkat T cells was induced by UV (254 nm) lamp for 15 min and incubated for another 4 hours at 37°C with 5% CO_2_. For the generation of apoptotic neutrophils, peritoneal neutrophils from C57BL/6 mice were cultured in complete RPMI for 24 hours.

***In Vitro* Phagocytosis Assays.**

Mouse peritoneal macrophages were isolated from 10 to 12 weeks old female mice, 72 hours after intraperitoneal injection of 2 ml of 3% Brewer’s thioglycolate (Sigma-Aldrich Corp, Darmstadt, Germany). Primary peritoneal macrophages were plated in 6-well plates in DMEM with 10% FBS and allowed to rest overnight at 37°C with 5% CO_2_, before the start of experiments. The non-adherent cells were removed.

Bone marrow-derived macrophages were generated from bone marrow progenitor cells from the 10-12 weeks old female mice. Freshly prepared bone marrow cells were cultured in DMEM with 10% FBS in the presence of 50 ng/ml M-CSF (Sigma-Aldrich Corp, Darmstadt, Germany) for 7 days, the non-adherent cells were removed on day 7, the adherent macrophages were transferred to 6-well plates for the experiment.

Before being fed to macrophages, apoptotic cells were labelled with pHrodo™ Green (Thermo Fisher Scientific, Carlsbad, CA, USA), a pH-sensitive phagocytosis-dependent indicator that requires no wash steps or quenchers, according to the manufacturer’s instructions. Unless specifically noted, peritoneal macrophages were incubated with pHrodo-labelled apoptotic cells at a ratio of 1:5 (macrophages: apoptosis cells) and cultured at 4°C (negative control) or 37°C for 60 min in RPMI supplemented with 10% FBS. In some cases, macrophages were pre-treated with 6-AN (10 μM, Sigma-Aldrich Corp, Darmstadt, Germany), DHEA (1μM, MedChemExpress, Monmouth Junction, NJ, USA) or AG1 (3μM, MedChemExpress, Monmouth Junction, NJ, USA) for 24 hours before co-incubation with pHrodo-labelled apoptotic cells. After incubation, cells were resuspended and stained with fluorescence-labelled anti-F4/80 antibody (BM8, BioLegend, San Diego, USA); they were later analysed by flowcytometry. Doublet discrimination was used to distinguish internalized from externally bound apoptotic cells and the proportion of macrophages containing ingested apoptotic cells was determined.

**Flow Cytometry.**

Single-cell suspensions were washed twice in staining buffer, resuspended, and incubated with anti-CD16/32 antibodies (Sungene Biotech，Tianjin, China) to block Fc receptors (0.5 μg/million cells). Then, the cells were subjected to staining with labelled antibodies diluted in staining buffer for 20 min at 4°C. After incubation, cells were washed in staining buffer and analysed immediately. For all staining, isotype controls were used. Following staining, cells were washed and suspended in PBS and then analysed on CANTO II (Becton Dickinson, Sunnyvale, CA, USA). The following labelled Abs were used: anti-F4/80 antibody (BM8, BioLegend, San Diego, USA), anti-CD68 antibody (FA-11, Sungene Biotech，Tianjin, China), anti-annexin V antibody (Sungene Biotech, Tianjin, China). Flow data were collected with CellQuest Software and analysed with FlowJo software.

**RNA-Sequencing.**

*In vitro* cultured WT or *Dicer1-CKO* mice peritoneal macrophages were incubated with apoptotic human Jurkat T cells at a ratio of 1:5 (macrophages: apoptosis cells) for 6 hours. Following washing, cells were cultured for another 4 hours and then total RNA was extracted using the Trizol kit (Invitrogen, Carlsbad, CA, USA) according to the manufacturer's protocol. RNA sequencing was performed using the Illumina HiSeq2500. Differentially expressed genes were performed by DESeq2 (PMID: 25516281) software between the two groups. Genes with a false discovery rate below 0.05 and an absolute fold change of ≥ 1.5 were considered differentially expressed genes. We performed a gene set enrichment analysis using the software GSEA to determine if a set of genes in specific pathway showed significant differences in the two groups. Briefly, we entered the gene expression matrix and ranked the genes by the Signal2Noise normalization method. Enrichment scores and p-values were calculated under default parameters.

**ELISA and biochemical parameters.**

The presence of anti-dsDNA antibodies in serum was tested using Mouse Anti-dsDNA IgG ELISA Kit (Alpha diagnostics, Santa Monica, CA, USA), per manufacturer’s protocol. The presence of ANA in serum was tested using Mouse ANA/ENA IgG ELISA Kit (Alpha diagnostics, Santa Monica, CA, USA), according to manufacturer’s protocol. The absorbance at 450 nm was measured using a Paradigm Multi-Mode Plate Reader (Beckman Coulter, Fullerton, CA, USA). Similarly, the presence of NADPH and glutathione in macrophage was examined using a NADPH ELISA Kit (Beyotime, Jiangsu, China) and glutathione ELISA Kit (Beyotime, Jiangsu, China) according to the manufacturer’s instructions. For the assessment of urine, mice were individually kept in sterilized metabolic cages and urine samples were pooled over 24 hours. The total urine protein was determined with a Urine protein test kit (Nanjin Jianchen, Jiangsu, China). The concentrations of creatinine and urea in the urine and serum samples were measured with the Creatinine Assay Kit (Nanjin Jianchen, Jiangsu, China) and Urea Assay Kit (Nanjin Jianchen, Jiangsu, China) according to the manufacturer’s instructions.

**Real-time quantitative PCR.**

Real-time PCR was performed as described. In brief, total RNA was harvested using RNAfast200 Reagent (Fastagen, Shanghai, China),or miRcute miRNA Kit（DP503.and total RNA was reverse transcribed into first-strand cDNA using cDNA reverse transcription kit (Takara, Shiga, Japan) or miRcute miRNA cDNA kit (Ribobio, Guangzhou, China). The cDNA was used to measure the relative expression of genes using Real-time PCR kits (Takara, Shiga, Japan) or miRcute miRNA Real-time PCR kits (Ribobio, Guangzhou, China) according to the manufacturer’s protocol. The mRNA levels were normalized to β-actin as an internal control. The gene expression values were expressed as the relative mRNA level (fold change) compared to untreated controls; whose expression levels were set as 1. The primers (Invitrogen, Carlsbad, CA, USA) used to measure the gene expression are listed as follows: β-actin (forward, 5′-TGGAATCCTGTGGCATCCATGAAA-3′; reverse, 5′-TAAAACGCAGCTCAGTAACAGTCCG-3′), Dicer (forward, 5′- GAAGACGTTCATCGCGGTC -3′; reverse, 5′- GCTGACACTTGTTGAGCAACC -3′), IL-1β (forward, 5′-GAAATGCCACCTTTTGACAGTG-3′; reverse, 5′- TGGATGCTCTCATCAGGACAG -3′), IL-6 (forward, 5′- TGATGGATGCTACCAAACTGG -3′; reverse, 5′-TGGTCTTGGTCCTTAGCCACT-3′), IL-10 (forward, 5′-GCTGGACAACATACTGCTAACC-3′; reverse, 5′- ATTTCCGATAAGGCTTGGCAA -3′), TNFα (forward, 5′- AACTAGTGGTGCCAGCCGAT -3′; reverse, 5′- CTTCACAGAGCAATGACTCC -3′), TGF-β (forward, 5′-CCACCTGCAAGACCATCGAC-3′; reverse, 5′- CTGGCGAGCCTTAGTTTGGAC-3′), G6pdx (forward, 5′-CACAGTGGACGACATCCGAAA-3′; reverse, 5′- GCAGGGCATTCATGTGGCT -3′), Pgd (forward, 5′-AAGCTGACATTGCACTGATCG-3′; reverse, 5′- CGGCGGGGCTTCTTTAGTT -3′), H6pd (forward, 5′-AGTGGAGGACTATCAGACCCT-3′; reverse, 5′- GGCGGCACACTGAAGTAGAAG -3′), Tkt (forward, 5′-ATCACAGCCTCAAGTCAGTGG-3′; reverse, 5′- TTCAGGTCATCGGGTTGCAC -3′), Taldo1 (forward, 5′-GTGGGGCGCATCCTTGATT-3′; reverse, 5′- TGGTCTTGTAGCCGAACTTCT -3′), Aldoc (forward, 5′-TGTACCGCCAGGTCCTATTCA-3′; reverse, 5′- GAGGCACTACACCCTTGTCAA -3′), Pfkl (forward, 5′-GGAGGCGAGAACATCAAGCC-3′; reverse, 5′- GCACTGCCAATAATGGTGCC -3′).

**Western blot analysis.**

Western blot’s samples were homogenized in ice-cold RIPA buffer with 1 mg/mL protease inhibitor cocktail. The homogenates were centrifuged at 13,500 rpm for 5 min at 4°C and the supernatants were collected. BCA assays were subsequently performed to determine the protein concentration of the supernatants. Cell extracts were resolved by sodium dodecyl sulfate–polyacrylamide gel electrophoresis (SDS-PAGE) and electrophoretically transferred onto PVDF membranes (Immobilon-P, Millipore). The following antibodies were used: Dicer (Abcam, Cambridge, MA, USA), GAPDH (Abcam, Cambridge, MA, USA) and goat antirabbit IgG-HRP secondary antibody (Novus Biologicals, Littleton, Colorado,USA). Proteins were visualized with an enhanced chemiluminescence system (Beyotime, Jiangsu, China). Results are representative of at least 3 independent experiments.

**Histological assessment.**

Histopathologic exami­nation of the skin, lung, spleen, and kidney samples was performed as previously reported. Briefly, tissues were harvested, fixed in 4% paraformaldehyde, dehydrated, bisected, and mounted in paraffin and sectioned for HE staining according to the manufacturer’s protocol. For immunohistochemical staining, paraffin-embedded tissue blocks were cut into 4-μm sections. After dewaxing, the sections were boiled (in a 600 W microwave oven) in citrate buffer (2.1 g sodium citrate/L, pH 6) for 15 min. The sections were cooled to room temperature and endogenous peroxidase was inhibited with 1% hydrogen peroxide (H2O2) in methanol for 15 minutes. To block the non-specific binding of immunoglobulins, sections were incubated with 3% albumin bovine V. After that, the sections were incubated with primary antibody at 4°C overnight.

The following antibodies were used: C3 (Abcam, Cambridge, MA, USA), goat anti-mouse IgG biotin secondary antibody (Abcam, Cambridge, MA, USA), goat anti-rat IgG biotin secondary antibody (Abcam, Cambridge, MA, USA) and goat anti-rabbit IgG-biotin secondary antibody (Abcam, Cambridge, MA, USA). After washing, the sections were incubated with corresponding secondary antibodies for 30 min. Subsequently, the Vecta-stain ABC kit (Vector Laboratories, San Diego, CA, USA) was used for the avidin–biotin complex method according to the manufacturer’s protocol. Peroxidase activity was visualized with a DAB Elite kit (K3465, DAKO, Copenhagen, Denmark). The sections were lightly counterstained with hematoxylin and dehydrated through an ethanol series to xylene and mounted. TUNEL staining (Derma TACS, Trevigen Inc., Gaithersburg, MD, USA) was performed to identify apoptotic cells in sections from the spleen, skin, and kidney with the following procedure: 4 μm sections were fixed with 2% PFA, permeabilized with 0.1% Triton X-100 in 0.1% sodium citrate, and stained with the TUNEL reaction. All sections were viewed using a light or fluorescence microscope.

**Statistical analysis.**

Data from at least three independent experiments were calculated with a statistical soft­ware package (GraphPad Prism 9). Two groups were analysed by the two-tailed unpaired Student’s t-test, and multiple groups were compared by one-way or two-way ANOVA with Tukey’s post hoc test for multiple groups. For all statistical analyses, statistical significance is indicated by a single asterisk (p-value < 0.05), two asterisks (p-value < 0.01), three asterisks (p-value < 0.001) and four asterisks (p-value < 0.0001).

**Data and software availability.**The RNaseq datasets generated from current study are available at NCBI: PRJNA694927 (https://www.ncbi.nlm.nih.gov/sra/PRJNA694927 ).
